# Toward designing human intervention studies to prevent osteoarthritis after knee injury: A report from an interdisciplinary OARSI 2023 workshop

**DOI:** 10.1016/j.ocarto.2024.100449

**Published:** 2024-02-23

**Authors:** Jackie L. Whittaker, Raneem Kalsoum, James Bilzon, Philip G. Conaghan, Kay Crossley, George R. Dodge, Alan Getgood, Xiaojuan Li, Elena Losina, Deborah J. Mason, Brian Pietrosimone, May Arna Risberg, Frank Roemer, David Felson, Adam G. Culvenor, Duncan Meuffels, Nicole Gerwin, Lee S. Simon, L. Stefan Lohmander, Martin Englund, Fiona E. Watt

**Affiliations:** a Department of Physical Therapy, University of British Columbia, Vancouver, Canada; b Arthritis Research Canada, Vancouver, Canada; c Department of Immunology and Inflammation, Imperial College London, London, UK; d Department for Health, University of Bath, Bath, UK; e Centre for Sport, Exercise and Osteoarthritis Research Versus Arthritis, UK; f Leeds Institute of Rheumatic and Musculoskeletal Medicine, University of Leeds, Leeds, UK; g NIHR Leeds Biomedical Research Centre, Leeds, UK; h La Trobe Sport and Exercise Medicine Research Centre, School of Allied Health, Human Services and Sport, La Trobe University, Melbourne, Australia; i Department of Orthopaedic Surgery, Perelman School of Medicine, University of Pennsylvania, Philadelphia, PA, USA; j Mechano Therapeutics LLC, Philadelphia, PA, USA; k Division of Orthopedic Surgery, Bone and Joint Institute, Fowler Kennedy Sport Medicine Clinic, Schulich School of Medicine and Dentistry, University of Western Ontario, London, ON, Canada; l Program of Advanced Musculoskeletal Imaging (PAMI), Cleveland Clinic, OH, USA; m Department of Biomedical Engineering, Lerner Research Institute, Cleveland Clinic, OH, USA; n Department of Orthopedic Surgery, Brigham and Women's Hospital, Boston, USA; o Department of Orthopedic Surgery, Harvard Medical School, Boston, USA; p Biomechanics and Bioengineering Research Centre Versus Arthritis, School of Biosciences, Cardiff University, Cardiff, UK; q Department of Exercise and Sport Science, University of North Carolina, USA; r Norwegian School Sport Sciences, Oslo, Norway; s Division of Orthopedic Surgery, Oslo University Hospital, Oslo, Norway; t Department of Radiology, Universitätsklinikum Erlangen & Friedrich- Alexander-Universität (FAU) Erlangen-Nürnberg, Erlangen, Germany; u Chobanian & Avedisian School of Medicine, Boston University, Boston, MA, USA; v Section of Rheumatology, Boston University Chobanian & Avedisian School of Medicine, Boston, MA, USA; w Orthopedic and Sport Medicine Department, Erasmus MC, University Medical Center Rotterdam, Rotterdam, the Netherlands; x Novartis BioMedical Research, Basel, Switzerland; y SDG LLC, Cambridge, MA, USA; z Department of Clinical Sciences Lund, Orthopaedics, Lund University, Lund, Sweden; aa Department of Clinical Sciences Lund, Orthopaedics, Clinical Epidemiology Unit, Lund University, Lund, Sweden; ab Centre for Osteoarthritis Pathogenesis Versus Arthritis, Kennedy Institute of Rheumatology, University of Oxford, UK

**Keywords:** Knee, Osteoarthritis, Post-traumatic osteoarthritis, Prevention, Randomised controlled trials, Trial design

## Abstract

**Objective:**

The global impact of osteoarthritis is growing. Currently no disease modifying osteoarthritis drugs/therapies exist, increasing the need for preventative strategies. Knee injuries have a high prevalence, distinct onset, and strong independent association with post-traumatic osteoarthritis (PTOA). Numerous groups are embarking upon research that will culminate in clinical trials to assess the effect of interventions to prevent knee PTOA despite challenges and lack of consensus about trial design in this population. Our objectives were to improve awareness of knee PTOA prevention trial design and discuss state-of-the art methods to address the unique opportunities and challenges of these studies.

**Design:**

An international interdisciplinary group developed a workshop, hosted at the 2023 Osteoarthritis Research Society International Congress. Here we summarize the workshop content and outputs, with the goal of moving the field of PTOA prevention trial design forward.

**Results:**

Workshop highlights included discussions about target population (considering risk, homogeneity, and possibility of modifying osteoarthritis outcome); target treatment (considering delivery, timing, feasibility and effectiveness); comparators (usual care, placebo), and primary symptomatic outcomes considering surrogates and the importance of knee function and symptoms other than pain to this population.

**Conclusions:**

Opportunities to test multimodal PTOA prevention interventions across preclinical models and clinical trials exist. As improving symptomatic outcomes aligns with patient and regulator priorities, co-primary symptomatic (single or aggregate/multidimensional outcome considering function and symptoms beyond pain) and structural/physiological outcomes may be appropriate for these trials. To ensure PTOA prevention trials are relevant and acceptable to all stakeholders, future research should address critical knowledge gaps and challenges.

## 1 Introduction

Osteoarthritis (OA) affects more than 14% of the world's population [[1], [2], [3]] and is a leading cause of pain, disability and socioeconomic costs [1,2]. The burden of OA is well established and expected to continue to grow [4]. There are no disease modifying drugs (DMOAD) or therapies (DMOAT) to combat OA [5]. This leaves prevention as the primary means available to curb the increasing global impact of OA [6]. The field of OA prevention is relatively young [7].

**Fig. 1:**
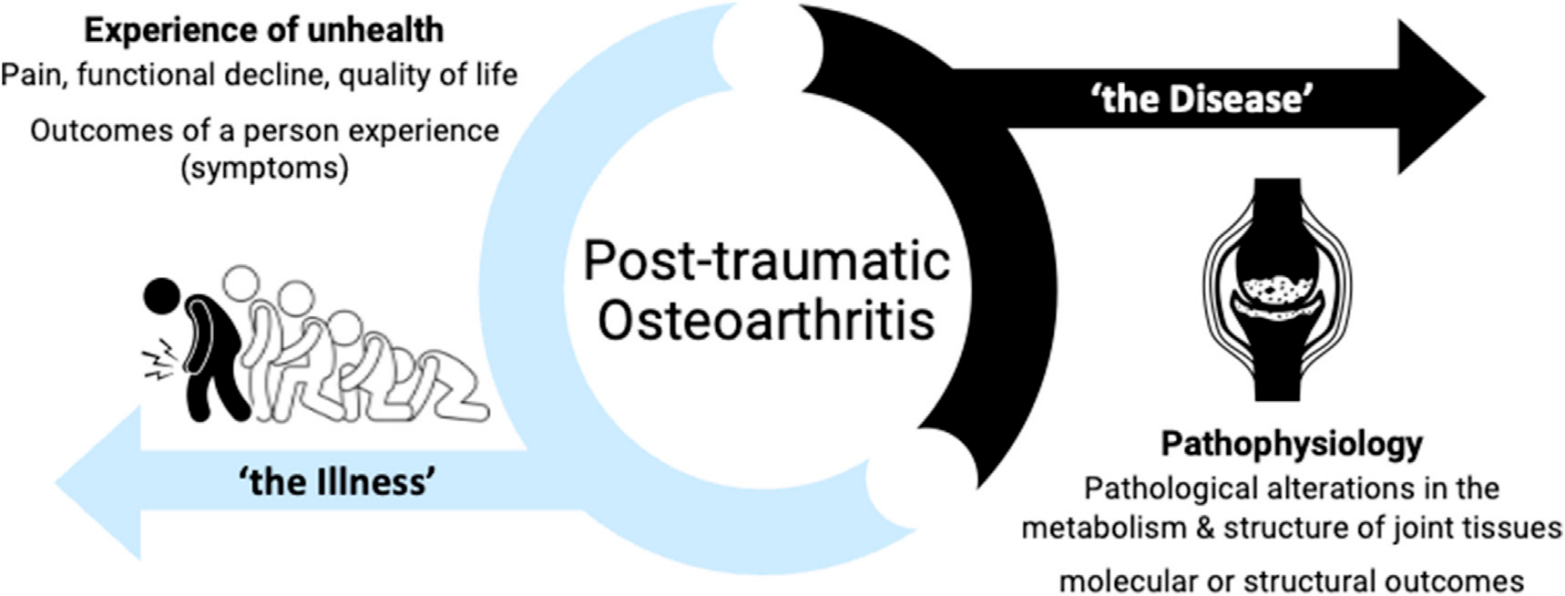
Post-traumatic osteoarthritis, as both a disease and an illness.

Traumatic knee injury has emerged as an attractive prevention target given it is highly prevalent, has a distinct onset and a strong independent association with future post-traumatic OA (PTOA) [15], which accounts for at least 12% of OA cases globally (i.e., 36 million people) [3]. PTOA can be viewed as both a disease (pathophysiology measured with molecular and structural outcomes) and an illness (experience of unhealth measured as symptoms including pain, functional decline and reduced quality of life; Fig. 1) [7].

Globally, scientific groups are embarking upon research programs that will culminate in prevention clinical trials targeting people at elevated risk of knee OA following knee injury (i.e., secondary prevention) [[8], [9], [10]]. Common examples of knee injuries include anterior cruciate ligament (ACL) ruptures and acute traumatic meniscal tears. However, there are significant challenges associated with the design of randomised controlled trials (RCTs) seeking to assess the effect of preventative interventions in these populations, and no consensus in the scientific community about how to approach these studies [7].

In an effort to improve awareness of PTOA prevention RCT design and the unique opportunities and challenges presented by seeking to prevent knee PTOA, an international organising group met over an 11-month period and developed a workshop entitled ‘*Designing human intervention studies to prevent osteoarthritis after knee injury: an interdisciplinary workshop’.* This workshop was hosted during the 2023 Osteoarthritis Research Society International (OARSI) Congress on March 17, 2023, Denver, Colorado, US. This paper synthesises the outputs of this process with the goal of moving the field of OA prevention trial design forward.

## 2 Purpose and learning objectives

**Fig. 2:**
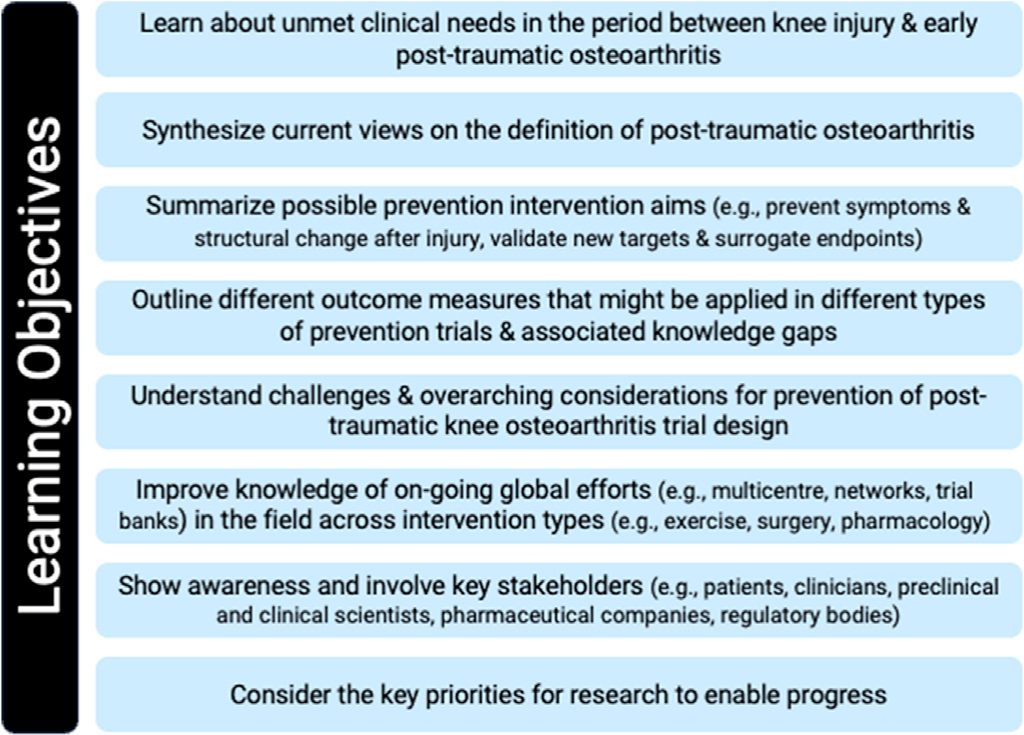
Workshop learning objectives.

The workshop's overarching aims were to increase awareness, present the current state-of-the-art on trial design, review challenges, and inform a research agenda for designing interventional trials preventing knee PTOA. The *‘a priori’* learning objectives for the workshop were purposefully multifaceted (Fig. 2).

## 3 Organizing group and workshop scope

Experts from the knee injury, OA, and prevention fields who represented multiple disciplines and diverse perspectives were invited to join an organising group led by FW to provide insights into the potential challenges associated with knee PTOA prevention trials. This 18-person organising group (spanning biomedical, clinical and health services research) and diversity of age, career stage, gender, culture, and country developed the workshop program. A proposal was submitted to an open call by OARSI for pre-congress workshops. The speaker list and program were finalized by the organising group in conjunction with the OARSI board and congress program committee. The 150-min pre-congress workshop was open to all delegates attending the 2023 OARSI annual congress in-person.

**Fig. 3:**
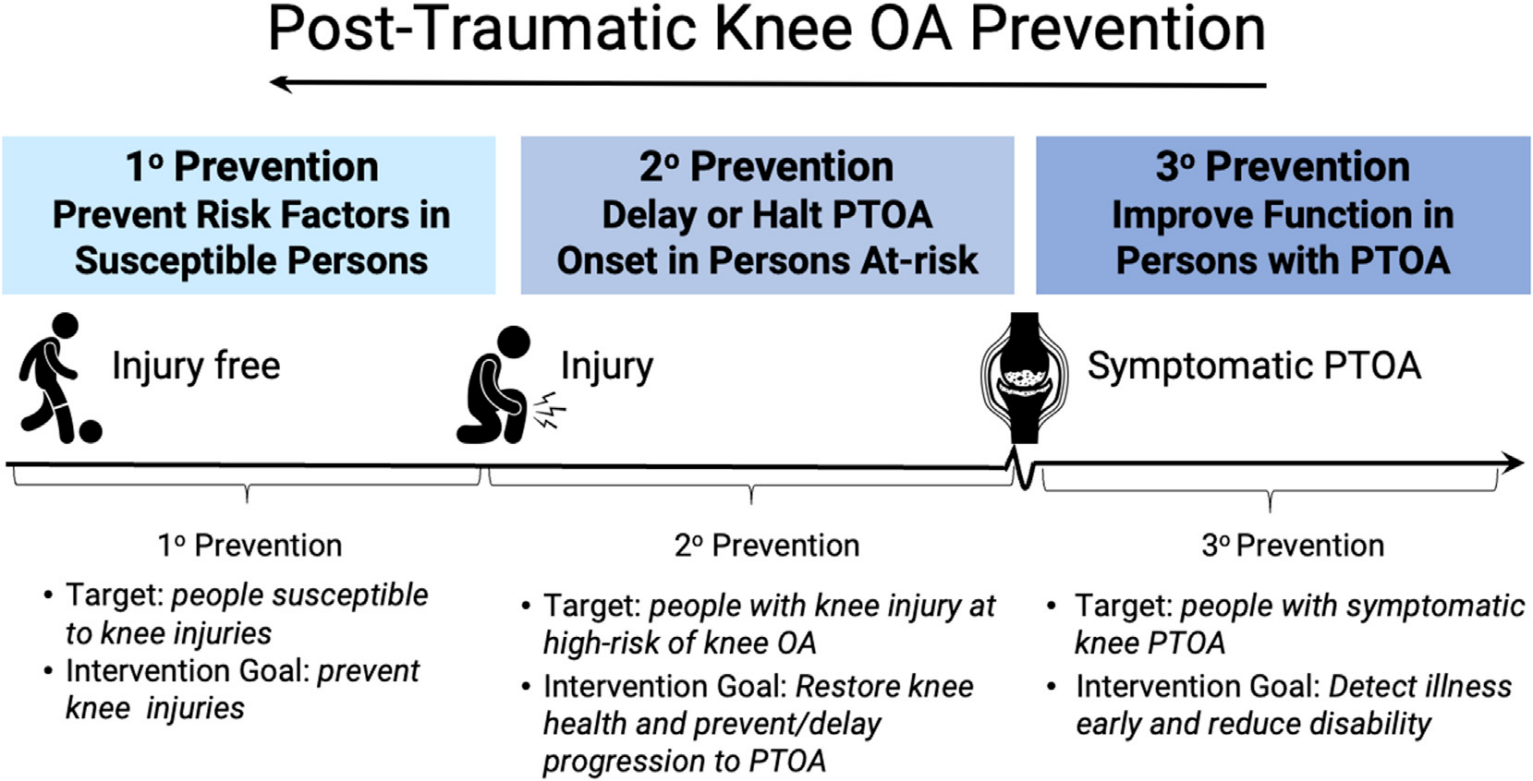
Levels of post-traumatic knee osteoarthritis (PTOA) prevention. This workshop was focussed on trials intervening to achieve secondary prevention of PTOA, i.e., at the time of, or after the knee injury occurring.

The workshop focused on human studies aiming for secondary prevention (halting, delaying, or reducing symptomatic OA severity after risk factor exposure) of OA after knee injury (Fig. 3). This focus acknowledged that learnings can be taken from pre-clinical and translational models and that knee joint injuries account for the bulk of evidence and majority of the health burden of PTOA (∼83%) [11]. The program consisted of three themed sessions identified by the organising group. Each session included a clarifying question period, with a longer moderated (FW, ME) discussion at the end of the workshop. No pre-workshop assignment or materials were provided. The workshop was not recorded but detailed notes were taken, compiled and reviewed by the speakers and organising group which directly informed this report.

## 4 Workshop program

The co-chairs (FW, ME) welcomed attendees and reviewed the workshops' priorities and learning objectives. The ethos for the event was introduced, centered around disciplines and attendees: (1) learning from each other's experience, (2) recognizing the possibility of more rigorous and impactful studies by harmonizing study design and reporting (accepting it is unlikely that one size fits all), and (3) working together to overcome key challenges, barriers, and knowledge gaps for the design and delivery of prevention RCTs.

### 4.1 Workshop sessions

The first session ‘*Preventing post-traumatic OA illness and disease: vision and challenges’* consisted of two presentations. The first, entitled *‘What is PTOA, who develops it and what is the goal of an intervention?’* (JW) addressed the definition of PTOA or what are we trying to prevent; what prevention entails or what we are trying to do, and; who develops knee PTOA or who should we target with prevention trials. The second presentation, entitled ‘*Defining the best outcome measures, their timing and relationships: symptoms, structure and molecules’* (SL) focused on candidate trial outcome(s) or endpoint(s) as they relate to measuring OA illness and disease, including surrogate outcome(s) or endpoint(s). See Table 1 for an overview of the speaker and key presentation points for this session.

**Table 1:** Preventing post-traumatic osteoarthritis illness and disease: vision and challenges presentation topics, speakers, and key points.

Topic and speaker	Overview of key presentation points
*What Is PTOA, who develops it and what is the goal of an intervention?* Dr. Jackie Whittaker, PT, PhD, Associate Professor, University of British Columbia, Canada	What is PTOA? • PTOA is both a disease (pathophysiology measured with molecular and structural outcomes) and an illness (experience of unhealth measured as symptoms-pain, functional decline and quality of life). • OA illness, not disease, drives the burden of OA (i.e., pain and functional decline cause people to seek healthcare, take sick leave and retire early, and pain is associated with early mortality). • To reduce the burden of OA it is illness (e.g., pain and functional decline) that must be prevented or reversed not necessarily pathophysiological changes. What is Prevention? • There are three opportunities to prevent knee PTOA. • Primary prevention refers to strategies that prevent knee injuries in people who are susceptible or have a high exposure. • Secondary prevention refers to strategies that identify injury early and restore knee health to delay or halt progression to knee OA in people at greatest risk. • Tertiary prevention refers to strategies that improve function and reduce disability in people who have knee PTOA. • This workshop focuses on secondary prevention. Who to Target with Prevention Trials • A recent systematic review and meta-analysis summarized who is most likely to progress to symptomatic and structural knee OA after an injury [17]. • Symptomatic OA (illness) risk is greatest in people with a ACLR and concomitant injury or ACLR in conjunction with a medial meniscectomy, and/or who have higher BMI 2-years after ACL surgery (repair or reconstruction). • Structural OA (disease) risk is greatest in people with a PF dislocation±a chondral lesion, ACL tear±a concomitant injury, or stand-alone meniscal tear, fracture, TF dislocation and recurrent PF dislocation. • The ideal controlled clinical prevention trial study population would be people with a homogenous injury that is a strong risk factor for knee PTOA, highly prevalent, and easy to detect. Accordingly, the most suitable target group are people with ACL tears and concomitant injuries. OPTIKNEE • JW highlighted the OPTIKNEE consensus group which recently published 7 systematic reviews [17,27,[38], [39], [40], [41], [42]] and a consensus paper [28] with clinical and research recommendations for preventing knee PTOA as a resource for attendees.
*Defining the best outcome measures, their timing and relationships: symptoms, structure and molecules* Dr. L. Stefan Lohmander, MD (Orthopaedic Surgery), PhD, Professor Emeritus, Lund University, Lund, Sweden	Clinical Trial End Points/Outcomes • Endpoints are an objectively measured outcome or event that can be used to determine whether an intervention is beneficial. • Clinical endpoints are characteristics or variables that measure how a patient feels, functions or survives. Surrogate End Points/Outcomes • Surrogate endpoints are defined as markers (e.g., molecule, imaging feature, physical sign) that are thought to predict but do not measure clinical benefit. They are characterized by their level of clinical validation. • ‘Validated’ surrogates predict or correlate with a clinical benefit. • ‘Reasonably likely’ surrogates correlate with a clinical benefit but lack sufficient clinical data to be ‘validated’. • ‘Candidate’ surrogates are still under evaluation for their ability to predict clinical benefit. We currently have possible imaging and molecular candidate surrogates for OA disease. • ‘Biomarker’ surrogates are indicators of a biological or pathological process, or response to a therapeutic intervention. Suggested Candidate Outcomes for Prevention Trials • *Primary outco*me: a single clinical or ‘illness’ endpoint (how person feels, functions and survives) based on a PRO (i.e., KOOS4, IKDC, or WOMET). • *Co-primary or secondary outcome(s)*: meniscal, cartilage, and/or bone (osteophytes, lesions or shape) structural endpoint based on x-ray, MRI (conventional or 3D), CT or USI. • *Secondary and exploratory illness outcomes*: measures of pain (NRS, ICOAP) health-related quality of life (SF-36/SF-12), physical activity, sports participation, social roles, functional performance, anxiety and depression, and adverse events/harms. • *Secondary or exploratory disease outcomes*: a ‘set’ of molecular markers. • When choosing outcomes, consider multiplicity, accuracy, standardization, diagnostic accuracy, validation, clinical utility, ease of implementation, harmful effects and possibility for classification bias. • If FDA/EMA approval will be sought, include regulators early in the process.

ACL (Anterior cruciate ligament), ACLR (ACL reconstruction), CT (Computerized Tomography), EMA (European Medicines Agency), FDA (Food and Drug Administration), ICOAP (Intermittent and constant OA pain score), IKDC (International knee documentation committee), KOOS_4_ (weighted average of four Knee injury and osteoarthritis outcome score subscales; pain, symptoms, function in sport and recreation, knee-related quality of life), MRI (magnetic resonance imaging), NRS (numerical rating scale), OA (osteoarthritis), PF (Patellofemoral), TF (Tibiofibular), PRO (patient-reported outcomes), PTOA (post-traumatic osteoarthritis), SF-36 (36-Item Short Form Survey), SF-12 (12-Item Short Form Survey), USI (ultrasound imaging), WOMET (Western Ontario Meniscal Evaluation Tool).

The second session entitled *‘Bridging the gap to clinical trials’* also consisted of two presentations. The first, entitled *‘Considerations for PTOA: A regulatory perspective’* (LS) addressed how therapeutics are approved in the USA including concept endpoints for confirmatory clinical trials in OA, the difference between real-world data and real-world effect, and considerations for prevention trial outcome selection considering current regulatory models. The second presentation, entitled *‘Can pre-clinical models and experimental medicine studies help intervention selection and trial design?’* (NG), covered the most commonly used pre-clinical models in PTOA, current understanding of the pathomechanisms underlying PTOA based on pre-clinical models, strengths and limitations of these models, and future opportunities for experimental medicine and early phase human clinical trials. See Table 2 for an overview of the speaker and key presentation points for this session.

**Table 2:** Bridging the gap to clinical trials presentation topics, speakers, and key points.

Topic and speaker	Overview of key presentation points
*Considerations for PTOA: a regulatory perspective* Dr. Lee Simon, MD (Rheumatology), Former Division Director of the FDA Analgesic, Anti-inflammatory, Ophthalmologic Drug Products Division, Cambridge Massachusetts, USA	DMOAD/DMOAT Approval • Regulators apply the ‘disease modifying’ label based on the totality of accumulated evidence about the balance of benefit to harm. This ensures stakeholders (e.g., patients, healthcare providers) can understand the therapeutic benefits and harms. • Benefit refers to a clinically relevant/meaningful change in how a patient feels, functions or survives replicated in ≥2 adequately powered RCTs. • Surrogate endpoints ‘reasonably likely’ to predict how a patient feels, functions or survives may be used for accelerated approval followed by a post-approval confirmatory study demonstrating clinical relevance. Challenges for Prevention DMOAD/DMOATs • As there is no benefit to harm calculation for approval of a prevention DMOAD/DMOATs, any therapeutic must be extraordinarily safe. Current Status of DMOAD/DMOATs • The FDA recently acknowledged that moderate to severe symptomatic OA is a ‘serious disease’ and the importance of identifying a window to introduce DMOADs/DMOATs early to alter its natural history. • There are no defined surrogate measures for OA. • The FDA recently (2020) proposed a composite endpoint for OA including TKA and symptoms (i.e., pain and function) that would require a 3-year study with up to 18,000 participants. Using Real-world Data to Generate Real-world Evidence • RWD are data about patients' health status and/or health care delivery routinely collected from a variety of sources. • RWE is clinical evidence about the use and potential benefits or harms of a therapeutic derived from RWD analysis. • The strength of RWE to support a DMOAD/DMOAT application depends on the study design (i.e., required randomization) study methodology, data reliability (need for data quality control and assurance specifically when combining data from multiple sites), and data relevance. ProActive • LS introduced ‘ProActive’; a public private partnership with the FDA for developing new and improved clinical trial outcomes which might be relevant for knee PTOA.
*Can Pre-clinical Models and Experimental Medicine Studies Help Intervention Selection and Trial Design?* Dr. Nicole Gerwin, PhD, Director, Immunology Disease Area at Novartis BioMedical Research, Basel, Switzerland	Using Pre-clinical Models to Inform Intervention Selection and Trial Design • Pre-clinical rodent models lead to rapid OA pathology (weeks) compared to human disease (10–15 years). • Surgically induced PTOA models (medial meniscus or/and ACL transection) are widely used to evaluate pharmacological interventions. • Non-invasive traumatic injury models (externally-applied load to induce ACL tear, cyclic tibial compression) more closely mimic human PTOA (pronounced inflammation and concomitant tissue injuries) and are used to study pathomechanisms and pharmacological interventions. • Pre-clinical model outcomes typically include histopathology (structural damage of joint tissues, cartilage loss, subchondral bone remodelling, osteophyte formation, synovial inflammation) and pain Current Understanding of PTOA Pathomechanisms, Therapeutics and Future Directions • Acute (≤2-months) PTOA pathomechanisms include: mechanical overload-induced cell necrosis and damage/loss of ECM molecules; hemarthrosis which activates neutrophils and mononuclear cells leading to apoptosis, tissue damage and impaired joint lubrication; prominent inflammation producing oxygen free radicals, inflammatory mediators, matrix degrading enzymes leading to apoptosis, and synovium/capsule fibrosis. • Chronic (10–15 years) PTOA pathomechanisms include: persistent and progressive joint tissue damage through apoptosis and ECM degrading enzymes; slow resolution of inflammation, and; altered biomechanical forces that promotes joint tissue degeneration. • Therapeutic trials in pre-clinical PTOA models have evaluated inhibition of inflammation, reactive oxygen species production, chondrocyte hypertrophy and bone turnover, and induction of cartilage regeneration. • DMOADs being explored include: anti-inflammatories (cytokine inhibitors - IL1Ra, anti-IL1, anti-IL17, anti-NLRP, IL-10), anti-catabolics (cartilage degrading enzymes inhibitors - ADAMTS-5 inhibitors, MMP inhibitors), and cartilage regeneration anabolics (LNA043, Lorecivivint, Sprifermin). • Future steps are to evaluate efficacy of the most promising candidates, alone and combined treatments in non-surgical preclinical PTOA models, and determine the optimal intervention timing for each pathomechanism. Advantages of Pre-Clinical Models • Pre-clinical PTOA models allow assessment of therapeutic efficacy (alone or in combination) within weeks, and at early and consistent time points after injury. • Pre-clinical PTOA models can speed the discovery of DMOADs by selecting candidate interventions and pharmacodynamic markers for clinical evaluation, and provide ideas for optimal intervention timing post-injury and treatment regimen. Potential of human experimental medicine studies • Clinical experimental PTOA studies offer the opportunity to identify biomarkers of PTOA development and imaging endpoints for clinical trials, and pharmacodynamic markers of candidate activity if combined with pharmacological treatment.

ACL (anterior cruciate ligament), ADAMTS (a disintegrin and metalloproteinase with thrombospondin motifs), DMOAD (disease modifying OA drug), DMOAT (disease modifying OA therapy), ECM (Extracellular Matrix), FDA (Food and Drug Administration), IL1Ra (Interleukin-1 receptor antagonist protein), IL1 (Interleukin-1), IL-10 (Interleukin 10), LNA043 (modified, recombinant version of the human angiopoietin-like 3), MMP (Matrix metalloproteinases), NLRP (Nod-like receptor protein), OA (Osteoarthritis), PTOA (post-traumatic osteoarthritis), RWD (Real World Data), RWE (Real World Evidence), TKA (Total Knee Arthroplasty).

Finally, the third session entitled ‘*From around the real world: Current examples of trials, their interventions, and comparators’* consisted of three presentations by speakers who gave examples of current prevention trials purposefully spanning, exercise-based, surgical and pharmaceutical interventions. The first, entitled *‘Exercise/Physical Therapy (SUPER-Knee)’* (AC), presented a rationale for exercise as a therapeutic for knee PTOA prevention and overviewed the ongoing SUpervised exercise-therapy and Patient Education Rehabilitation (SUPER)-Knee trial [10]. The second presentation, entitled *‘Surgical (ROTATE-Trial, COMPARE)* (DM) discussed the basis for choosing orthopaedic surgery or exercise-based rehabilitation as therapeutics for knee PTOA prevention. This was followed by an overview of the Study of Traumatic meniscal tears: Arthroscopic Resection vs Rehabilitation (STARR) [12], Conservative vs Operative Methods for Patients with ACL Rupture Evaluation (COMPARE) [13], and Rupture Of The Anterior cruciaTe ligamEnt - an algorithm study (ROTATE) [14] trials. Finally, the third presentation, entitled ‘*Pharmacological (OACTN Initiative)*’ (DF) proposed a method of participant selection for testing pharmacology agents for knee PTOA prevention [15], and introduced the Arthritis Foundation's Osteoarthritis Clinical Trials Network (OACTN) Initiative. See Table 3 for an overview of the speaker and key presentation points for this session.

**Table 3:** From around the real world: Current examples of trials, their interventions and comparators presentation topics, speakers, and key points.

Topic and speaker	Overview of key presentation points
*Exercise/Physical Therapy (SUPER-Knee)* Dr. Adam Culvenor PT, PhD, Senior Research Fellow, La Trobe Sport and Exercise Medicine Research Centre, La Trobe University, Melbourne, Australia	Rationale for exercise-based Interventions • Exercise enhances symptoms, function and physical activity after injury; improves pain and QoL for persons with OA, and; may alter PTOA risk. SUPER-Knee Trial (Trial number: ACTRN12620001164987) • *1° Objective:* compare the effect of a SUpervised exercise-therapy and Patient Education Rehabilitation (SUPER) versus a minimal intervention control on self-reported pain, function and QoL (KOOS4) in young adults with an ACLR. • *Design*: parallel-group, assessor-blinded, RCT. • *Sample*: 184 persons aged 18–40 years, 9–36 months post-ACLR with ongoing symptoms (KOOS4 <80/100) suggesting a need for treatment. • *Intervention*: 4-month individualized, PT-supervised strengthening and neuromuscular program (based on ACSM recommendations) with education. Months 0–4: 2 ​PT supervised and 1 unsupervised session/week. Months 5–12: unsupervised self-management with 2 booster sessions. • *Control*: minimal intervention (best-practice guide booklet and 1 face-to-face orientation appointment with a PT). • *1° Outcom*e *(illness)*: 4-month change in KOOS4. • *2*^*o*^*Outcomes (illness and disease)*: 4-month change in KOOS subscale scores, patient-perceived improvement (GROC), thigh muscle performance (isokinetic dynamometer), knee functional performance (hop battery) and MRI cartilage morphology (MOAKS) and composition (T2 mapping), and bone shape. • *Other Outcomes*: HRQoL (EQ-5D-5L), kinesiophobia (TSK), physical activity (Tegner, accelerometer), pain (NPRS), treatment adherence, other treatments, adverse events. • *Primary Analysis*: Fully powered intention-to-treat linear model adjusted for baseline measure and referral source (private vs public healthcare). • *Status*: In data collection [10]
*Surgical (STARR, COMPARE and ROTATE-Trials)* Dr. Duncan Mueffels, MD (orthopaedic surgery), PhD	Rationale for Comparing Surgical and Exercise-based interventions • Surgery and exercise-based rehabilitation improve symptoms, function, and PROs after meniscal lesion and ACL tear. It is not clear if one approach is superior or more appropriate for certain patient sub-groups. STARR Trial (Netherlands Trial registration number: NTR 4511) • *1° Obje*ctive: Compare the effect of arthroscopic partial meniscectomy or PT on self-reported symptoms and function in young people with traumatic meniscal tears. • *Design*: Open-labelled, multicenter, parallel group superiority RCT. • *Sample*: 100 persons, aged 18–45 years (mean 35.1 ​± ​8.1), with a recent traumatic MRI-verified, isolated grade 3 meniscal tear without knee OA. • *Intervention*: arthroscopic partial meniscectomy ​± ​post-operative PT. • *Control*: 3-month tailored standardized PT program (phase 1: reduce effusion; phase 2: optimize ROM and restore coordination/muscle function; phase 3: simulate activities for daily living and RTS) ​± ​arthroscopic partial meniscectomy after 3-months. • *1*^*o*^*Outcome (illness)*: 24-month IKDC score. • *2*^*o*^*Outcomes (illness and disease):* 24-month KOOS subscale scores, knee-related pain (NRS), symptoms (Lysholm) and QoL (WOMET), sporting level (Tegner), satisfaction with knee function (VAS), serious adverse events. • *Primary Analysis:* Fully powered intention to treat linear model adjusted for baseline measure, randomization type and surgeon. • *Results*: Early arthroscopic partial meniscectomy was not superior to a strategy of physical therapy with optional delayed arthroscopic partial meniscectomy at 24-month follow-up. • *Status*: Completed [12]. • *Learnings*: Patient recruitment was challenging of 196 eligible, 100 participated), patients had a preference to which treatment they wanted and doctors had an opinion about which patients were surgical candidates. COMPARE Trial (Netherlands Trial Register number: NL2618) • *1° Obj*ective: Compare the effect of early ACLR versus rehabilitation with optional delayed ACLR for patients with an acute ACL rupture on self-reported symptoms and function in and sports participation at 2-years. • *Design*: Open labelled, multicenter, parallel RCT. • *Sample*: 167 persons, aged 18–65 years (mean 31.3), with a recent (6-weeks) acute ACL rupture MRI-verified. • *Intervention*: early ACLR (<6-weeks) ​+ ​rehabilitation. • *Control*: 3-month rehabilitation as per the Dutch ACL guideline [43] with optional delayed (>3-month) ACLR. • *1° Outcome* (il*lness):* 24-month IKDC score. • *2° Outcomes* (illness and disease): 24-month KOOS subscale scores, knee-related pain (NRS) and symptoms (Lysholm), return to pre-injury sport level, giving way, sporting level (Tegner), treatment satisfaction, serious adverse events. • *Primary Analysis*: Fully powered intention to treat mixed model (restricted maximum likelihood approach) adjusted for baseline measure, follow-up period, sex, BMI and age. • *Results*: Early ACLR group had non-clinically relevant better outcome at 24-months than rehabilitation ​+ ​delayed ACLR group. The rehabilitation group had better outcomes up to 6-months. • *Status*: Completed [13]. • *Learnings*: 50% of the rehabilitation group went on to ACLR and it is unclear if they would have benefited from early ACLR. ROTATE Trial (Netherlands Trial registration number: NL8637) • *1° Object*ive: Compare the 2-year effect and cost-effectiveness of a treatment algorithm versus current care for primary ACL rupture patients. • *Design*: multicenter, open-labelled cluster randomized controlled trial with superiority design. • *Sample*: 200 people aged ≥18-years, with a complete, primary ACL rupture (MRI-verified) and maximum of 6-weeks of non-operative treatment. • *Intervention*: Treatment decision based on an algorithm (informed by COMPARE RCT and orthopaedic surgeons/researchers) that advises patients if they will respond to non-surgical (rehabilitation) and shared decision-making process. • *Control*: Treatment decision based on orthopaedic surgeon and patient preference. • *1*^*o*^*Outcome* (illness): 24-month IKDC score. • *2*^*o*^*Outcomes (illness and disease):* 24-month KOOS subscale scores, knee-related pain (NRS) and symptoms (Lysholm), kinesiophobia (TSK), HRQoL (EQ-ED-5L), return to pre-injury sport level, giving way, sporting level (Tegner), treatment satisfaction, serious adverse events, quality of shared decision making (SDM-Q-9), technology acceptance (surgeons), medical costs (iMCQ) and productivity loss (iPCO). • *Primary Analysis*: Treatment Effect (Fully powered intention to treat cluster (site) linear model adjusted for baseline measure and randomization allocation), cost utility (QALY) and cost effectiveness, and qualitative (shared-decision making) analyses will be performed. • *Status*: In data collection [14].
*Pharmacological (OACTN Initiative)* Dr. David Felson MD (rheumatology), MPH, Professor, School of Medicine, Boston University, Boston, Mass, USA	Rationale for Pharmacological Interventions • Currently there are no approved DMOADs, and DMOAD development has experienced many failures partly due to changes in the OA joint with time. • Barriers to DMOADs targeting PTOA prevention include lag time between injury and PTOA onset, and the relatively small number of people that develop PTOA after an ACLR. • A possible solution would be to test DMOADs in persons at high risk of rapidly developing OA or target OA in its early stages (this would also prevent pain induced nervous system changes). FASTOA • *Aim:* Identify patients at high risk of persistent knee pain after ACLR and high likelihood of developing early-onset OA to inform the conduct clinical trials to identify DMOADs (target population). • *Coordination*: FASTOA is coordinated by the AF OACTN (USA). • *Approach*: Using the MOON cohort (2800 ACLR patients with 2-year follow-up) investigators are assessing imaging and biomarkers that identify patients at high risk of persistent knee pain after ACLR at 2-years. • *Results*: 16.6% of ACLR patients had clinically significant pain (KOOS ≤80), and 26.3% had moderate knee pain on one activity at 2-years. Those at high risk of clinically significant knee pain were more likely to have higher baseline knee pain scores, be overweight and to have had a chondral injury or severe meniscal tear at the time of surgery. Older age, male sex and other meniscal tears were not associated with persistent pain after ACLR. PIKASO Trial • This is a planned multi-center RCT for patients who have had an ACLR and are at high risk for knee PTOA (clinically significant knee pain at baseline and chondral injury).

ACL (Anterior cruciate ligament), ACLR (ACL reconstruction), ACSM (American College of Sports Medicine, AF OACTN (Arthritis Foundation Osteoarthritis Clinical Trials Network), COMPARE (The Conservative versus Operative Methods for Patients with ACL Rupture Evaluation), DMOAD (Disease modifying Osteoarthritis Drugs), DM-Q-9 (Shared decision making questionnaire), EQ-5D-5L (5-level EQ-5D version quality of life questionnaire), GROC (Global Rating of Change Score), IKDC (International Knee Documentation Committee), HRQoL (Health-related quality of life), iMCQ (Medical Consumption Questionnaire), iPCO (Production Consumption Questionnaire), KOOS_4_ (weighted average of four Knee injury and osteoarthritis outcome score subscales; pain, symptoms function in sport and recreation, knee-related quality of life), MOAKS (MRI OA Knee Score), MOON (Multicenter Orthopedic Outcomes Network), MRI (magnetic resonance imaging), NRS (Numerical rating pain scale), OA (Osteoarthritis), PRO (patient-reported outcomes), PIKASO (Preventing Injured Knees from osteoArthritis Severity Outcomes), PTOA (Post-traumatic osteoarthritis), QALY (Quality-adjusted life year), RCT (Randomized controlled trial), ROM (Routine Outcome Measures questionnaire), ROTATE (Rupture Of The Anterior cruciaTe ligamEnt - an algorithm study), STARR (Study of Traumatic meniscal tears: Arthroscopic Resection vs Rehabilitation), TAM (technology acceptance model), TSK (Tampa Scale of Kinesiophobia questionnaire), VAS (Visual Analogue Scale), WOMET (Western Ontario Meniscal Evaluation Tool).

### 4.2 Workshop discussions

A summary of the key discussion points for individual sessions, and the overall workshop follow below.

#### 4.2.1 Preventing post-traumatic OA illness and disease: vision and challenges

The questions and discussion that followed the first session's presentations were related to the concept of PTOA illness (i.e., symptomatic OA) and if this is sufficient as a stand-alone outcome; what features might help to identify people most likely to progress to knee PTOA (target trial population); and the need for outcomes specific to the period between injury and OA onset. During the discussion several important concepts arose. First, that pain is not a primary complaint for people who have recovered from a traumatic knee injury, so may not be a responsive outcome. Alternatively, there are other symptoms (e.g., functional loss or lack of confidence in the knee) that are self-reported to be more important [16]. Second, injury and/or surgery type, health-seeking behaviours, and functional status are constructs that might be useful for identifying target trial populations. Current evidence [17] supports that people at greatest risk for progression of symptomatic and structural OA are those with ACL ruptures treated with reconstruction surgery (ACLR) who have concomitant chondral and meniscal lesions, or additional meniscal surgery. Less is known about the relationship between health-seeking behaviours or functional status and progression to knee PTOA. Third, that knee PTOA prevention trial outcomes need to be specific and sensitive to the period between injury and OA onset, as opposed to the period beyond OA diagnosis. This may require the development and testing of new outcomes and/or use of existing outcomes (i.e., IKDC, KOOS) currently accepted by regulators. These existing outcomes have excellent measurement properties in populations that span people living with various traumatic knee injuries, OA and total joint arthroplasty [18,19]. Finally, the discussion emphasized that an intervention's effect on outcomes of OA illness (e.g., how a patient feels, functions and survives) should be the focus of prevention RCTs but that outcomes of OA disease (e.g., structure, pathophysiology) may be important secondary outcomes or in some cases could be considered as a co-primary outcome, depending on the nature of the intervention. Table 1 in the Supplementary File provides a detailed summary of the questions and responses for Session 1.

#### 4.2.2 Bridging the gap to clinical trials

The questions and discussion that followed the second session's presentations were related to the challenge of assessing pain (illness) in animal (rodent) models. More specifically, that validated PTOA animal models tend to focus on structural (histology) outcomes while reliable and accurate pain or functional outcomes for animals that translate directly to human illness (e.g., nociceptive, nociplastic and neuropathic pain) are not straight forward. It was pointed out that structural indicators that correlate with pain such as histological inflammation scores, joint swelling and bone remodelling may usefully inform effects of interventions on human illness [20,21]. It was also highlighted that the field is rapidly evolving and there are emerging behavioral pain assessments and direct assays for pain responses and pathways (electrophysiology, anatomy, omics) [22]. Further, functional MRI can inform us about how the central nervous system is activated. Another key discussion point was that some preclinical research purporting to test the effect of a knee OA intervention, is actually testing PTOA prevention (i.e., the intervention is administered prior to, or around the time of injury) so may be highly relevant to the field [23,24]. Table 2 in Supplementary File provides a detailed summary of the questions and responses for Session 2.

#### 4.2.3 From around the real world: current examples of trials, their interventions and comparators

The questions and discussion that followed the third session's presentations related to challenges encountered when conducting rigorous RCTs in real world settings; the importance of monitoring adiposity after injury; the goal(s) of prevention interventions, and; similarities (or differences) between fast progressing traumatic and non-traumatic OA. Real world challenges discussed included the methods and feasibility of selecting and recruiting a homogenous yet representative sample of people that are likely to progress to PTOA, and screening for structural injury status (e.g., presence or absence of concurrent meniscal and osteochondral injury) prior to enrollment. It was recognized that body mass, and perhaps more specifically fat mass, are important considerations in this population, as injury and prolonged recovery can impact activity levels and result in a vicious cycle of weight gain. With that said, accurate assessment of fat mass is instrument-based (e.g., dual X-ray absorptiometry) which can be costly and increase participation burden which is why it is often not included. One key concept discussed was that the goal (and related design) of prevention intervention trials might not necessarily be to ‘halt’ the onset of PTOA, but rather to ‘flatten the slope’ or ‘delay’ and ‘reduce’ the severity of PTOA so that patients experience as few symptoms and disabilities as possible for as long as possible (decrease the number of ‘young people with old knees’) [25]. Finally, the differences between people with rapidly developing traumatic and rapidly progressing non-traumatic OA were highlighted, with suggestions that they may be fundamentally different sub-groups with different underlying processes at play (definitive evidence is lacking). Table 3 in the Supplementary File provides a detailed summary of the questions and responses for Session 3.

#### 4.2.4 Final group discussion

After the last presentation session, all speakers participated in a broader panel question and answer session, with members of the convening group and audience asked to contribute. The questions that arose during the overall discussion were related to the optimal timing of pharmaceutical interventions after injury, including those that are targeting inflammation, and the best outcomes to measure OA illness in persons with a past ACL rupture, including performance-based and muscle function outcomes. The discussants acknowledged that while ACLR may benefit some, it induces a second insult to the knee joint [26], and it could be beneficial to target aspects of the injury response such as inflammation prior to surgery as part of a complex intervention design. It was also highlighted that orthopaedic surgeons would welcome discovery of new methods to reduce the trauma associated with an ACLR.

Another key theme in the conversation was the importance of selecting a clinically meaningful endpoint for prevention trials that is relevant to participants and clinicians (i.e., OA illness endpoint). In the post-ACLR population this may need to extend beyond pain to include constructs such as knee-related quality of life, the ability to participate in sport and recreational activities, social consequences of injury, fear of re-injury and overall knee satisfaction. It could also be appropriate to measure performance-based and knee muscle (e.g., strength) function, even though regulators have not historically been supportive of function as a primary outcome. It was highlighted that muscle function testing can be resource-intensive as it requires in-person data collection, instrumentation and skilled assessors. Also discussed was the lack of an agreed upon muscle function outcome [27], and limitations of comparing between legs (central changes) or to an often-unknown pre-injury value that may have been suboptimal and predisposed to the injury occurrence. Table 4 in the Supplementary File provides a detailed summary of the questions and responses for the overall discussion.

**Table 4:** Knowledge gaps that impede post-traumatic knee osteoarthritis prevention trials design and delivery.

Aspect of trial^a^ design	Area	Gap
Population	Eligibility	What participant characteristics/features/markers are best to consider when including participants in trials?
	Stratification	Should participants be stratified? Should stratification be done at enrollment or after? Should stratification be by participant characteristics (e.g., sex) or factors related to risk of OA, or intervention responsiveness?
Intervention	Timing	What is the best approach to define the window of opportunity for any given intervention?
	Delivery	How is the acceptability (participants and providers) of an intervention (including dose and delivery mode) determined?
	Underlying mechanism	What are the mechanisms underlying the beneficial effect of interventions (e.g., exercise and lifestyle change, surgery, pharmaceutical) on pain, symptoms and function in persons at increased risk of knee PTOA?
Comparator	Usual care	What is the definition of usual care for people at risk of OA after a traumatic knee injury? How does usual care vary internationally (and what is the best way to cater to this)?
	Placebo	What is a credible placebo condition (controls for natural history, regression to the mean and contextual effects including attention, without offering a treatment effect) for education and exercise-based interventions?
	Other confounders	What is the best way to control for other factors that confound the intervention and outcome relationship (e.g., participant characteristics that change over time, other interventions)?
Outcome	Primary symptomatic outcome	What is the optimum single symptomatic outcome (including composite outcomes) for use in trials that is reliable, valid, sensitive to change, meaningful to patients and clinicians, and acceptable to regulators?
	Surrogate outcomes	What is the optimal method to define the transition from joint injury to early OA (relevant to eligibility criteria, end-point(s)) to ensure surrogate outcome(s) correlate with the stages of this transition appropriately?

OA (osteoarthritis), PTOA (post-traumatic osteoarthritis).

a The term trial throughout this table refers to secondary PTOA prevention trials.

## 5 Discussion

### 5.1 Workshop themes and future directions

Across the presentations and discussion during the workshop, several high-level themes emerged relating to knee PTOA prevention RCT endpoints, target population, and intervention design.

The most consistent and emphasized theme was that prevention trials must prioritize a clinically important symptomatic endpoint, which is demonstrated to be a robust surrogate for OA illness. Stated another way, an outcome that a patient (and regulator) will perceive as meaningful (i.e., the effect of the intervention on how the patient feels, functions and survives). This means that OA illness should be the target of the prevention intervention [7,28]. It was also acknowledged that understanding and measuring the underlying pathophysiology (OA disease) is important. A note of caution was that investigators in preclinical, translational and clinical fields should avoid assuming that modifying pathophysiology or symptoms alone would be the only thing that drives a person's experience. Practically, this might lead to a primary illness outcome and either a co-primary or secondary disease outcome. With respect to what the ideal illness outcome is for this population, it was highlighted on multiple occasions that pain is not a prominent complaint for people in the years following a traumatic knee injury and that it will be important to consider other symptoms, function and knee-related quality of life [16]. This suggests that a single aggregate/multidimensional patient-reported outcome (e.g., KOOS_4_, IKDC, WOMET) may be most relevant. There may also be relevant pathophysiology outcomes for this population such as structural changes identified through imaging or molecular biomarkers, including those in development. Translational research bridging animal models and human studies may have an important role in further identifying markers of pathophysiology.

Efforts to develop a robust clinical definition of ‘early-stage symptomatic knee OA’ for application to clinical trials are underway [29]. It was noted that a ‘early-stage symptomatic knee OA’ definition must include ‘early-stage symptomatic PTOA’ as there is currently no evidence that PTOA and ‘non-traumatic’ OA differ, other than in diagnostic considerations on MRI interpretation at early points after an injury. This would entail not including age as a diagnostic criterion as seen with OA classification criteria [30] given that knee PTOA commonly presents at a relatively young age. Having an agreed ‘early-stage OA’ outcome (or surrogate), that is valid for individuals being enrolled into PTOA prevention trials is critical for success. Preclinical models where pathophysiology can be followed from injury to established PTOA may help to identify early molecular indicators or predictive biomarkers of PTOA.

Another important theme that arose during the workshop was that the leading target population for prevention trials are people who have experienced an ACL rupture, considering the relative prevalence, homogeneity of injury type, ease of identification and healthcare impact. Based on the existing evidence [17] this could be further narrowed down to individuals with ACL rupture and a concomitant injury (i.e., meniscal or osteo-chondral lesion) or those treated with an ACLR and/or other surgery (i.e., partial or total meniscectomy). It is also possible, although not yet supported by causal evidence, that those who have persistent knee symptoms, a higher body weight or fat mass (either at the time of injury, or in response to the injury), poorer knee function, or who are less physically active (either at the time of injury, or in the longer term) may progress to PTOA more rapidly. These points are relevant for trial inclusion criteria, but also as important factors which could confound outcomes if not considered carefully in trial design.

The heterogeneity of the ACL rupture population was also discussed. There were a number of calls during the workshop for methods to increase homogeneity in trial populations, based on either a specific injury type and/or other features associated with a high knee PTOA risk, balanced against the potential for reversible PTOA disease or illness (i.e., PTOA is not ‘inevitable’) and generalizability to a broader at-risk population. These considerations have direct implications for recruitment (i.e., feasibility) and the scientific and commercial case for intervention development in specific populations, particularly of relevance to pharmacological trials.

Two consistent messages that surfaced from the sharing of novel real-world approaches, were that PTOA prevention RCT design is challenging, not only because there is a considerable time lag between injury and OA onset, but because individual OA risk varies considerably and treating clinicians and patients often have strong beliefs about what the best intervention is. These factors can interfere with recruitment and treatment fidelity. There is emerging evidence that not all people with injury benefit from any one type of intervention (e.g., exercise-based, surgery, pharmaceuticals) [31], and that in the real world several intervention types are typically combined (initially and over time). It is important to not only identify who is most likely to benefit from a particular intervention to facilitate individualized care (personalised medicine), but consider combinations of interventions in trial design (or at least take into account and control for aspects of usual care).

### 5.2 Knowledge gaps and areas for future research

The organising group noted a number of critical gaps in our current knowledge. These gaps need to be addressed to enable secondary knee PTOA prevention RCTs at scale as an active area of OA research, rather than a niche, high-risk activity which is not recognized by pharma industry or regulators. These gaps were compiled following iterative review by the organising group after the workshop and are outlined in Table 4. Though not exhaustive, this list represents key areas where we believe further evidence and guidance is needed. This knowledge is critical for many aspects of study methodology and would directly benefit efforts to design high quality RCTs with maximum chance of success (where success is a new intervention with regulatory or other relevant approvals, allowing implementation and adoption into routine practice internationally).

On reflection it is also important to highlight that some noteworthy topics were not discussed during the workshop. This included the involvement of people with lived experience of knee injury and/or knee PTOA in prevention trial design, to maximise study feasibility and acceptability. Similarly, the role of feasibility [32], proof-of-concept [33] or experimental medicine studies to aid definitive effectiveness trial design (or hybrid effectiveness and implementation designs [34]) to reduce the ‘evidence to practice gap’ were not explored. To overcome the disproportionate impact of knee OA experienced by some social groups it will be important to better understand the role of social determinants of health, including sex and gender, as they relate to risk and outcomes for knee joint injury [35,36] and PTOA [37] so that targeted interventions can be developed. Other important topics that did not garner much detailed discussion were novel imaging, biomechanical or molecular-based assessments, either to identify trial target populations or as early (surrogate) outcomes.

## 6 Summary

The workshop was met with a great deal of optimism by the audience, speakers and organising group regarding the potential for design and delivery of PTOA prevention trials. There was widespread recognition of the significant opportunities associated with preventing knee PTOA particularly the ability to identify and test interventions in preclinical models, creating opportunities for true bench-to-bedside translation.

Joint pain may not be the best symptomatic primary-endpoint/outcome for knee PTOA prevention RCTs and it will be important to consider other symptoms and functional outcomes. Aggregate or multidimensional outcomes may offer advantages. Targeting and measuring symptomatic PTOA outcomes would align with patient priorities and regulator needs based on experience of successful labels for OA and other aligned conditions. But this should not ignore the potential positive (or negative) effects on underlying disease process outcomes. An interdisciplinary approach was considered essential in developing the field successfully.

## Author contributions

FW conceived the idea of the workshop and worked with others in the organising group to develop the bid, agenda and workshop. JW, RK and FW drafted the manuscript. All authors (organising group members and invited speakers) reviewed the minutes from the workshop and contributed to and reviewed the manuscript prior to submission.

## Funding

FW is a recipient of a fellowship from UK Research & Innovation, a UK government supported research which includes the Medical Research Council (MR/S016538/1, MR/S016538/2 and MR/Y003470/1). This fellowship directly supported FW and RK in their time in coordinating all aspects of this work and in the writing of the manuscript. JW is supported by an Arthritis Society (Canada) STARS career development award (STAR 19–0493) and Michael Smith Health Research BC Scholar award (SCH-2020-0403). AC is a recipient of a National Health and Medical Research Council (NHMRC) of Australia Investigator Grant (GNT2008523). PC is supported in part by the United Kingdom National Institute for Health and Care Research (NIHR) Leeds Biomedical Research Centre. The views expressed are those of the authors and not necessarily those of the NHS, the NIHR, or the Department of Health. The Centre for Sport, Exercise and Osteoarthritis Research Versus Arthritis (funded by Versus Arthritis, 21595) covered limited speaker travel costs associated with the workshop.

Funding sources had no role in the review or contents of this manuscript.

## Organising/convening group affiliations

James Bilzon (representing CSEOA Research Versus Arthritis).

Philip Conaghan (PC) (representing ARUK∗ OA CSG expert working group).

Kay Crossley (KC) (representing OPTIKNEE, SEPA).

Adam CulvenorΨ (AC) (representing OPTIKNEE, SEPA), George Dodge (GD) (representing OARSI Strategic Alliance Committee).

Martin Englund (ME) (representing OARSI and ORS and member of ARUK∗ OA CSG expert working group).

David Felson (DF) (representing AF OACTN, ARUK∗ OA CSG expert working group).

Nicole Gerwin (Novartis).

Alan Getgood (AG) (representing AOSSM, ISAKOS, ACL study group).

Stefan Lohmander (representing OARSI Classification of Early OA taskforce; OPTIKNEE; ARUK∗CSG expert working group, CSEOA Scientific Advisory Board).

Xiaojuan Li (XL) (representing AF OACTN).

Elena Losina (EL) (representing AF OACTN).

Deborah Mason (DJM) (representing BORS pre-clinical science; ICORS; ARUK∗ OA CSG expert working group; Versus Arthritis Research Advisory Group).

Duncan Meuffels (DM) (representing Dutch Sport Orthopaedic Association and the Dutch Arthroscopic Society; ISAKOS).

Brian Pietrosimone (BP) (representing AF OACTN).

May Arna Risberg (MR) (representing OPTIKNEE, SEPA; CSEOA Scientific Advisory Board).

Frank Roemer (FR) (representing ARUK∗ OA CSG expert working group).

Lee Simon (representing SDG LLC).

Fiona Watt (FW) (representing NIHR MSK TRC, ARUK∗ OA CSG expert working group; Centre for OA Pathogenesis Versus Arthritis).

Jackie Whittaker (representing OPTIKNEE, SEPA, and Arthritis Research Canada).

AF OACTN (Arthritis Foundation Osteoarthritis Clinical Trials Network); AOSSM (American Orthopaedic Society for Sports Medicine); ARUK OA CSG (Arthritis Research UK (now known as Versus Arthritis) OA and crystal diseases Clinical Study Group); BORS (British Orthopaedic Research Society); CSEOA (Centre for Sports Exercise & Osteoarthritis); ICORS (International Combined Orthopaedic Research Society); ISAKOS (International Society of Arthroscopy, Knee Surgery and Orthopaedic Sports Medicine); OARSI (Osteoarthritis Research Society International); OPTIKNEE (International consensus group for optimizing knee health after injury to prevent knee osteoarthritis); ORS (Orthopaedic Research Society); SEPA (OARSI special interest group for osteoarthritis prevention related to Sport, Exercise, and Physical Activity).

∗ARUK, Arthritis Research UK, a UK Musculoskeletal charity now known as Versus Arthritis

ΨAC wishes to highlight that La Trobe University's SUPER-Knee program is not to be confused with the University of Newcastle's SuPeR Knee. Support. Predict. Recover® program (www.centrerehabinnovations.com.au).

## Declaration of competing interest

JW is a senior editor with the Journal of Orthopaedic and Sports Physical Therapy and associate editor with the British Journal of Sports Medicine.

AC is an associate editor of British Journal of Sports Medicine and Osteoarthritis and Cartilage.

FW is an associate editor of Osteoarthritis & Cartilage. In the last 3 years she has received consulting fees from Pfizer. FW is chair of the PARIS Trial Oversight Committee (unremunerated). FW is Co-lead, UK NIHR Translational Research Collaboration, Common MSK Conditions workstream (unremunerated).

FR is shareholder of Boston Imaging Core Lab (BICL), LLC. and consultant to Grünenthal. He is Editor in Chief of *Osteoarthritis Imaging* and Associate Editor of *Radiology*.

ME has received consulting fees from Cellcolabs AB.

NG is an employee and shareholder of Novartis Pharma AG.

DJM has patents granted for the use of drugs to prevent PTOA, has received research funding from Orphelion and tested drugs supplied by Orphelion and Novartis.

MAR is a Scientific Advisory Board member for Centre for Sport, Exercise & Osteoarthritis Research Versus Arthritis and a Board member of a non-profit Hospital in Oslo; Norway: Lovisenberg Rehabilitering.

PC has received consulting fees from AbbVie, AstraZeneca, Bristol-Myers Squibb, Eli Lilly, Galapagos, Genascence, GlaxoSmithKline, Janssen, Levicept, Novartis, Pfizer, Stryker and UCB.

GD has received consulting fees from Sanofi and Pacira and is owner/shareholder of Mechano Therapeutics LLC.

AG has received consulting fees from Smith and Nephew and owns shares/stock in Precison OS, Osteosys Robotics and LinkX Robotics.

SL has received consulting fees from Joint Academy and is member clinical trial DSMB, Astra Zeneca.

EL is on the OARSI board of directors and is the deputy editor of The Journal of Bone and Joint Surgery.
