# Supplementary material for: Toward designing human intervention studies to prevent osteoarthritis after knee injury: A report from an interdisciplinary OARSI 2023 workshop
Source: Osteoarthr Cartil Open. 2024 Feb 23;6(2):100449. doi: 10.1016/j.ocarto.2024.100449 (PMC10910316; doi:10.1016/j.ocarto.2024.100449)
Supplement: Multimedia component 1 [file mmc1.docx]

**SUPPLEMENTARY FILE**

**Table 1: Summary of individual questions and panel member responses for the ‘Preventing post-traumatic OA illness and disease: vision and challenges’ session**

| **Question/Comment** | **Response(s)** |
| --- | --- |
| 1. There’s a big conversation around illness vs disease and where both meet. Both talks suggest that we should be focusing on the illness, but is this sufficient enough and are there other aspects we need to consider? | - SL proposed that a sole focus on pain in this population is not helpful because it is often not their main complaint. Instead, there are other symptoms that these patients prioritize (e.g., functional loss, or a composite outcome including functional loss). SL supported the argument that patient reported outcomes (illness) that show the therapeutic has an impact on the patient experience should be prioritized as this is what the regulators require. - JW reiterated that the burden of OA is due to the illness and should be a key focus. JW also acknowledged that pathophysiology is undeniably part of the narrative and might be important secondary outcomes. Ultimately, we need to accept that it is patient experience that drives them to seek healthcare. |
| 1. Are there any considerations for pre-specified subgroups that you would suggest for clinical trials? | - JW acknowledged that not everyone who has a knee injury or ACL tear will develop PTOA. As trials are designed it is important to include people with the highest risk. Concomitant injuries are an important consideration with chondral lesions adding the most risk. Concomitant surgeries including meniscectomies might also be helpful. Another consideration that might be important is body mass index and/or and adiposity. |
| 1. Other than structural subgroups, could there be any personality traits, such as illness perception that could be relevant? | - JW suggested there could be, but none were identified in the systematic review. It is possible that traits related to healthcare seeking behaviours might be relevant but further evidence would be needed to determine this. |
| 1. Is there a need for the development of pre-OA outcomes? | - SL proposed this could be useful, but that the outcomes that already exist for OA are approved by regulators. If new outcomes are developed, they would need to be accepted by regulators. With that said, outcomes relevant to the pre-OA period might perform better. - JW added that many of the current OA outcomes have excellent measurement properties that span from time of injury through to living with OA. |
| 1. Should we include performance based functional outcomes in addition to patient reported function? | - JW agreed with this, and recognised that patient reported and performance-based outcomes of function (i.e. muscle strength & hop function) do not measure the same thing. Performance-based functional outcomes are seen as important secondary outcomes. JW also acknowledged that these outcomes could be a good way to identify PTOA risk (e.g., individuals with functional challenges) however to date there is insufficient evidence to support this. The challenges, although not insurmountable, with these outcomes is that they require in-person data collection, assessor training and some infrastructure, though some are possible with wearable technology. - SL re-iterated that patient reported and performance-based outcomes of function are not always related. |

ACL (Anterior cruciate ligament), OA (Osteoarthritis), PTOA (Post-traumatic osteoarthritis)

**Table 2: Summary of questions and responses for the ‘Bridging the gap to clinical trials’ session**

| **Question/Comment** | **Response(s)** |
| --- | --- |
| 1. The challenge faced with animal models is assessing pain aspect of the situation. It is easy to induce the pathology, but getting a pain assessment is difficult (they may be ‘stoic’). What is your opinion on the best pain assessment measure that is going to be the most reliable and potentially be predictive of a human situation? | - NG agreed that we do not have reliable, accurate pain assessments for animal models (especially rats) that are translational to humans and that ultimately, we require studies in humans to assess benefit on pain and other symptoms. NG also acknowledged it is very difficult to get an accurate measure of function although gait analyses during running is promising. - LS stated that there is a working group focussed on addressing the lack of translational evidence related to pain from animals to humans. Unfortunately, the situation has not improved in the last 20 years. It is possible that functional MRI imaging may translate to humans, however it is an expensive procedure. |
| 1. Rodent pain assessment is a rapidly evolving field. There are different pain assays that can be performed in rodents, which can inform us about different ways in which the nervous system is activated. It is also possible to perform non-behavioural assessments (i.e., direct assessments of pain pathways) to give us an understanding of how/which receptors have been activated, how the brain is responding using fMRI. Findings from behavioural and non-behavioural assessments appear to relate to different structural events occurring in the joint. It is important to keep performing & expanding on viable pain assessments. | - LS suggested we should be concerned about differentiating nociceptive, nocioplastic and neuropathic pain in animal models even though they all have a role in OA status and musculoskeletal pain. |

fMRI (functional magnetic resonance imaging), OA (osteoarthritis)

**Table 3: Summary of questions and responses for the ‘From around the real world: Current examples of trials, their interventions and comparators’ session**

| **Question/Comment** | **Response(s)** |
| --- | --- |
| 1. Question for DM. You mentioned that one exclusion criterion was a meniscal lesion that was eligible for a suture. This is something that is normally identified at the time of surgery. Did you exclude these patients after including them to the trial. If so, do you also think there could have been a bias here? | - DM stated that if it was thought that the meniscus lesion could be sutured based on examination of the MRI (acknowledging the lack of predictability), then patient would not be randomised. There were patients who had lesions that could be sutured, and they were sutured in the trial. If the patient had a locked knee, they were not eligible. |
| 1. Question for DM. Were the trials hard to run as they are focused on patient sub-groups? What are your thoughts on feasibility of stratification based on excluding 80% of eligible participants? | - DM stated that they were trying to get as homogenous of a group as possible but in doing so agreed that this does make the trial more difficult. DM felt that it would be more challenging to identify people who have persistent pain after ACLR and who meet the criteria for symptomatic OA as pain is not a real feature for this population. |
| 1. What are your thoughts on measuring fat mass and body mass index, as well as our need for better measures in trials? | - AC stated that BMI was discussed during the design of SUPER KNEE but it came down to feasibility. It was also thought that it would add an additional data collection burden for participants. AC shared that based on the baseline data, most of participants were overweight (mean BMI ~30kg/m^2^). With that said, most were fit and healthy before their injury, and that for many the injury led to inactivity and a vicious cycle with weight gain, suggesting that this is an important outcome. - DF agreed that fat mass is an important outcome, best measured with instrumentation (e.g., DXA) not BMI, and that it is always a balance between time, participant burden and expense. If BMI needs measuring, this could be self-reported and suit remote follow up. |
| 1. Are the selections we are making and environments we are creating too much of an uphill climb, or making it difficult for the treatment to work? (e.g., are some things associated with OA after injury inevitable). | - DF stated that we are not necessarily talking about halting OA, but rather delaying the process so it is less severe and/or the patient is more functional. The goal is to alleviate the extent of suffering (e.g., flatten the slope). - DM agreed and suggested that when patients seek ‘health care, they are ‘on the peak of the hill’ and as clinicians and researchers we have been successful in bettering their experiences and reducing their pain in some way. |
| 1. Question for DF. For primary OA (patients without a clear history of injury), a subgroup of patients also develops OA very fast. Do you think this can be categorised in the same way as PTOA, including the same risk factors and features? | - DF doubts that this could be the case and that the situation is not the same. Progressive OA is biomechanically and structurally driven and MRIs show irreversible changes in bone shape associated with synovitis. These people have multiple lesions, which makes it more difficult than in a post trauma situation. For those who have injury and subsequent PTOA some of the changes could be reversible. There is also the element of nervous system and pain channel changes that have not been discussed that occur in the peripheral nervous system and are present in a large percentage of people with chronic OA. These should not be present in ACLR patients as they are relatively acute. |

ACLR (Anterior cruciate ligament), BMI (Body mass index), DXA (Dual-energy x-ray absorptiometry), MRI (Magnetic resonance imaging), OA (Osteoarthritis), PTOA (Post-traumatic osteoarthritis)

**Table 4: Summary of questions and responses for the ‘Overall Discussion’ session**

| **Question/Comment** | **Response(s)** |
| --- | --- |
| 1. In terms of timing, when should pharmacological interventions be introduced? | - DF recognized the large increase in inflammation associated with ACLR as a rationale for introducing pharmacological prior to surgery. DM indicated it is ideal to recruit and randomise patients to the intervention and allow time for anti-inflammatory medications to take effect prior to ACLR. - AC reiterated that pain is not the main symptom for this patient population. Rather, it is difficulty participating in sport and recreational activities, and social consequences that are a priority. AG also indicated that fear of re-injury and lack of satisfaction with their knee is also prominent. For example, some participants report a KOOS_4_ score of 95, but report low knee satisfaction, while others have a KOOS_4_ of 50 but report satisfaction. AC proposed that the disconnect between pain and other symptoms are a rationale for a definition for symptomatic PTOA that is not just focused on pain. - DM stated that surgery can be beneficial at the time of injury, but he also performs surgery because the human body is sometimes unable to do things without a ‘push’. It is clear that surgery can increase inflammation and surgeons would appreciate methods to diminish the amount of trauma (and inflammation) associated with ACLR. |
| 1. The early stages of injury are different to OA yet we use the same pain outcome measures. As mentioned earlier, we should not limit our outcome to pain. There also seems to be a disconnect with how we measure drug efficacy, including in preclinical studies and how it is used in clinical trials. If we are unable to overcome this, then translation will never work. What are your opinions on this? | - JW stated that the KOOS_4_ is a good alternative to just focusing on pain because it incorporates other symptoms, knee-related QoL, and function in sport and recreation. - DF stated that they have looked at using different KOOS domains as well. DF suggested that imaging might help to understand what structure to target. There is no one answer to this dilemma but a good approach would be to measure both pain and structure. |
| 1. We heard about what regulators want and we were reminded that we should be aiming at PROs, but should this be pain or do we need to think about other domains in symptoms such as function? | - LS stated that the challenge with pain is that it is subjective and differs between people based on their experiences. - LS stated it has been argued that function is an important aspect of having a chronic disease, however regulators have not historically been interested in function as a primary outcome. With that said, we need to consider that function loss is important and to ask patients questions about what is meaningful to them. This means understanding from patients from what it means to ‘get better’. - LS stated that what DF is attempting to do (sampling from a real heterogenous population and explore how outcomes map on to how patients feel early on) is an important attempt to look at a unique patient population. This will help us to understand structure and symptoms. How the symptoms will be interpreted and what they mean will be a challenge. |
| 1. Do pharmaceuticals include natural products, or agents such as PRP? | - LS stated that RCTs for natural products are often poorly designed and do not provide good quality evidence. - LS spoke to a patient who told him they were cured from OA. - They paid $8000 for PRP treatment. When the patient was asked ‘what do you mean by you are cured’, they replied ‘I feel much better’. This showed LS that health related QoL is impacted by not just pain, but the ability to perform meaningful activities. - FW clarified that the focus of the workshop is trials of any types of interventions, which could in theory include natural products. |
| 1. We are talking about PTOA as a homogenous group when it is not, therefore we need to do more research in this area. We talk about pain and structure but not as much about muscle function, although this is important for patient’s ability to perform physical activity. | - AC stated that one of the secondary outcomes in SUPERKNEE is thigh muscle strength. - JW agreed that muscle function is a necessary outcome to bring into the conversation, but to consider that is it often not valued by regulators and is resource intensive (requires in-person data collection, instrumentation and skilled assessors). |
| 1. What is the best placebo control for an exercise intervention? | - ME suggested low dose exercise |

ACLR (anterior cruciate ligament reconstruction), KOOS (knee injury and osteoarthritis outcome score), KOOS_4_ (weighted average of four Knee injury and osteoarthritis outcome score subscales; pain, symptoms function in sport and recreation, knee-related quality of life), OA (osteoarthritis), PRP (platelet rich plasma), PRO (patient-reported outcome), PTOA (post-traumatic osteoarthritis), QoL (quality of life), RCT (randomized controlled trial)
